# Fully-spliced HIV-1 RNAs are reverse transcribed with similar efficiencies as the genomic RNA in virions and cells, but more efficiently in AZT-treated cells

**DOI:** 10.1186/1742-4690-4-30

**Published:** 2007-05-02

**Authors:** Laurent Houzet, Zakia Morichaud, Marylène Mougel

**Affiliations:** 1 CPBS, UMI, CNRS, 4 bd Henri IV, CS 69033, 34965 Montpellier, France

## Abstract

We have shown previously that HIV actively and selectively packages the spliced HIV RNAs into progeny virions. In the present study, by using a RT-QPCR and QPCR strategies, we show that spliced viral RNAs are present in infectious particles and consequently participate, along with the unspliced genomic RNA, to some of the early steps of infection such as the reverse transcription step. This work provides the first quantitative data on reverse transcription of the fully spliced viral RNAs, also called the early transcripts, in target cells but also inside virions. The latter results were obtained by measuring the natural endogenous reverse transcription activity directly on intact HIV-1 particles. Our study reveals that spliced HIV RNAs are reverse transcribed as efficiently as the genomic RNA, both in cells and virions. Interestingly, we also show that reverse transcription of spliced RNAs is 56-fold less sensitive to the inhibitor AZT than reverse transcription of the genomic RNA. Therefore, the selection mediated by inhibitors of reverse transcription used to treat patients could lead to increased representativeness of spliced forms of HIV, thus favoring recombination between the HIV DNA species and facilitating HIV recovery.

## Findings

HIV particles include two-copies of full-length genomic RNA (FL RNA) which represent less than 50% of the RNA mass in virions [1]. Indeed, HIV also packages viral spliced and cellular RNAs. Recently, in a detailed quantitative study, we showed that both singly and fully spliced viral RNAs are packaged with similar efficiencies into HIV-1 particles and by an active mechanism dependent of the FL RNA packaging [2]. Assuming that spliced HIV RNAs are packaged in infectious particles, we postulated that they are actively involved in some of the early stages of infection such as the reverse transcription (RTion) step. By using extensive QPCR strategies (well described in [2]), we investigated the fate of the spliced viral RNAs in HIV-1 infected cells, more specifically during RTion. We focused on the fully spliced (FSpl) RNAs because they represent the majority of the HIV-1 spliced transcripts and because they encode the regulatory proteins Tat, Rev and Nef, required to engage infection.

## Fully-spliced HIV RNAs are reverse transcribed as efficiently as the FL RNA within infected cells

**Figure 1:**
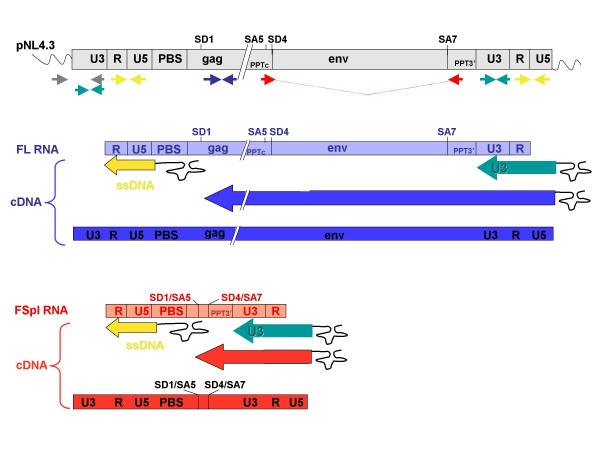
**Schematic representation of templates, primers and products of RTion reactions**. Only the 2 splice donor sites (SD1 and SD4) and the 2 splice acceptor sites (SA5 and SA7) important for this study are indicated. PCR-primer pairs used to specifically quantify the pNL4.3 plasmid (pNL), the FL cDNA or RNA (FL), and the Fspl cDNAs or RNAs (FSpl) are in gray, blue and red, respectively. Final proviral products corresponding to RTion of FL and FSpl RNAs are presented. RTion is a multi-step process and for clarity purposes, only the shortest intermediate RTion products detected with primer pairs specific for FL and FSpl are presented, as well as the shortest common ssDNA (yellow) and the U3 intermediate (green), respectively. All primer sequences and detailed PCR conditions will be provided on request.

Reverse transcription requires *cis*-acting elements of the FL RNA template including the Primer Binding Site (PBS) used for RTion initiation, the 5'R and 3'R regions, required for template switching, and the polypurine tracts (PPTc and PPT3') used for plus-strand DNA synthesis priming (Fig. 1) [3]. All these elements, except for the PPTc, are maintained within all spliced RNAs since the 5' major splice-donor site (SD1) is located downstream of the PBS (Fig. 1). This raises the possibility that virions-associated spliced RNAs may be reverse transcribed during viral replication.

**Figure 2:**
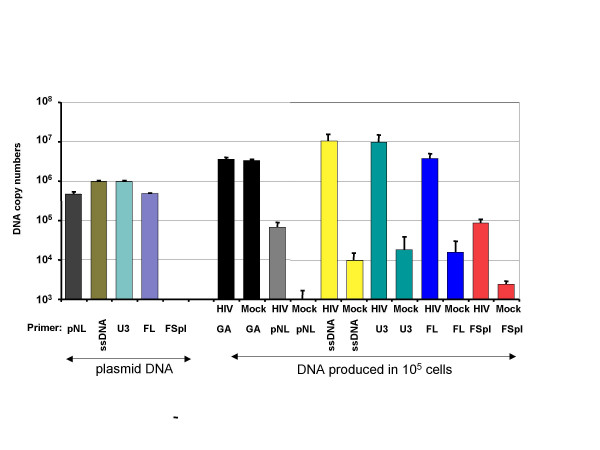
**Quantitative analysis of reverse transcription of spliced and unspliced HIV RNAs within infected cells**. The different RTion products were quantified in 25ng of cellular DNA samples and results derived from standard curves realized with pNL4.3 dilutions, were expressed as copy numbers (cps) in 10^5 ^cells. The GAPDH gene was monitored to control the input of cellular genomic DNA samples used in each assay. Reliability and specificity of the QPCR amplifications are illustrated with 5 × 10^5 ^cps of pNL4.3 plasmid. Since U3 and ssDNA targets occur twice in the pNL4.3 plasmid used for standard curve, the measured cps were multiplied by 2 to obtain the real number of target sequences in the corresponding sample. Results represent mean standard ± deviations of at least three independent experiments.

To address this issue, the stably CD4/CXCR4-coexpressing HEK-293 cells (42CD4) [4] were infected with HIV stocks produced by 293T cells previously transfected with the pNL4.3 plasmid [2]. After 24 h of infection, total cellular DNA was extracted and subjected to specific QPCR analysis. Under these settings, the effects of potential reinfection on RT efficiencies are not significant. First, we used primers allowing detection of the viral cDNA species from the earliest steps of RTion, *i.e*. the minus strong-stop DNA synthesis (ssDNA) and the first strand transfer (represented by the U3 product) (Fig. 1). Up to now, there is no data available on spliced viral RNA RTion efficiency in HIV-1 infected cells. To specifically quantify the overall "FSpl cDNA" forms we used a primer pair (FSpl) specific for the SD4/SA7 exon-exon junction [2] (Fig. 1). For comparative purposes, production of proviral DNA (FL) was also measured with a primer pair (FL) targeting the *gag *gene. All these primer pairs gave similar PCR reliability when used with pNL4.3 plasmid as a control template (Fig. 2). The plasmid DNA contamination arising from the pNL4-3 transfection used to produce virion stocks, was monitored by using a primer pair (pNL) specific for the pNL4.3 plasmid but not for HIV FL proviral DNA (Fig. 1) and was less than 2% (Fig. 2). Control amplifications on mock-infected cells with primer combinations specific for the ssDNA, U3, FL or Fspl RNAs gave only low unspecific background signal (Fig. 2). As expected, proviral DNA (FL) was mainly amplified in DNA extracted from infected cells (3.8 × 10^6 ^cps) since virions majoritarily contained FL RNA. Since the amounts detected with either the ssDNA or the U3 primers were close to 2-times of those measured with the FL primers, they mostly correspond to the entire FL cDNA. However, additional cDNA species corresponding to the RTion of FSpl RNAs, and presumably to the entire spliced proviral forms as previously shown by [5], were detected in infected cells and were 44-fold less abundant than FL cDNA (Fig. 2).

**Figure 3:**
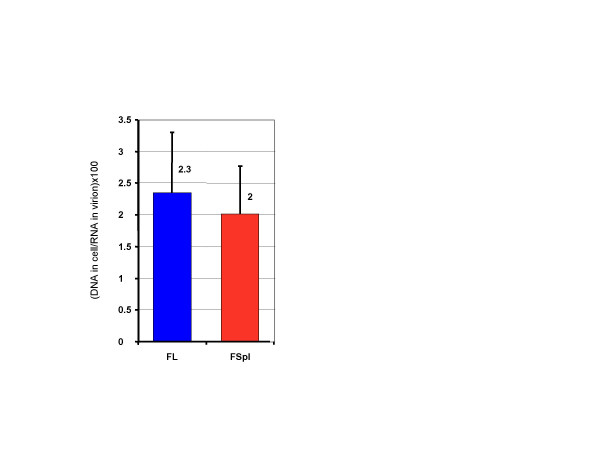
Apparent RTion efficiencies of FL and FSpl RNAs in HIV-1 infected cells.

To determine the RTion efficiencies of the FL and FSpl RNA species, we also quantified the corresponding RNA levels contained in the infecting virus stocks. The pNL4-3 copy numbers were subtracted from the FL DNA amount and we calculated the ratios of DNA amount in cells/RNA template level in infecting particles (Fig. 3). These results show that FL and FSpl RNAs are both packaged in infectious HIV particles and subsequently reverse transcribed in infected cells with similar efficiencies, suggesting that the HIV RTion machinery does not discriminate between these two templates and that their RTion is regulated by the same mechanism.

## Reverse transcription of spliced RNAs is less sensitive to AZT than RTion of the FL RNA

**Figure 4:**
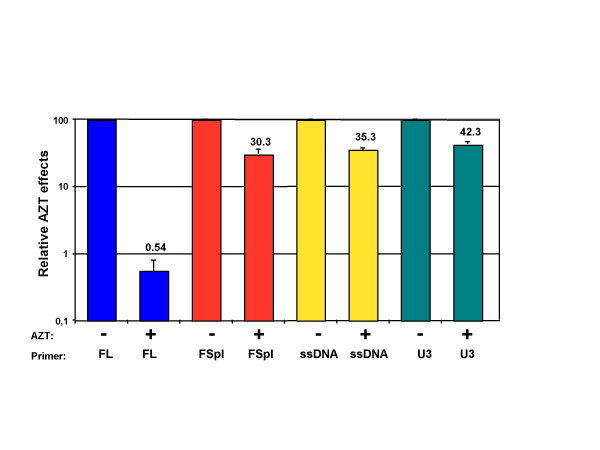
**Relative effects of AZT on different RTion products within infected cells**. The copy numbers (cps) were measured in 25ng of DNA extracted from cells treated or not with AZT and ratios were calculated as: (+AZT/-AZT) × 100. For each cDNA species, levels were average (± SD) from at least three independent experiments and normalized to level measured without AZT.

Reverse transcription of FL RNA is inhibited in the presence of AZT, an inhibitor of the reverse transcriptase (RT) widely used in the clinic for the treatment of HIV infection. AZT, after conversion to AZT-5'-triphosphate by cellular enzymes, inhibits RT through a competition with the natural dTTP [6]. To further investigate the RTion of spliced RNAs, we examined its sensitivity to AZT (Fig. 4). The 42CD4 cells were pretreated 3 h before infection with 10 μM AZT and infection was pursued for 24 h in the presence of 10 μM AZT. After DNA extraction, cDNA was quantified as before. As expected, the AZT treatment induced a strong decrease (185-fold) of the FL cDNA amounts in infected cells. In contrast, AZT induced only a 3-fold decrease of the "spliced cDNA" synthesis (Fig. 4). At least two non-exclusive hypothesis could explain this resistance of FSpl RTion to AZT, either the reduced number of AZT sites within short template or the achievement of RTion before the cell entry, as NERT, then escaping the AZT treatment.

The mechanism of AZT inhibition has been well studied both in vitro [7] and in infected cells [8], showing that inhibition occurred most efficiently when the DNA products of RT reaction were long. Note that FSpl RNAs are more than 4-fold shorter than FL RNA. We also measured the effects of AZT on intermediate-length reverse-transcribed DNA products, such as the minus ssDNA and U3 cDNA (Fig. 1). As shown in Fig. 4, and similarly to "Fspl cDNA", AZT has only little effect on the synthesis of these two short RTion intermediates.

This result suggests that in the infected cells exposed to AZT, the high level of the "FSpl cDNAs" is a direct consequence of their short length.

## Reverse transcription of FL and FSpl RNAs is achieved equally in intact virions

**Figure 5:**
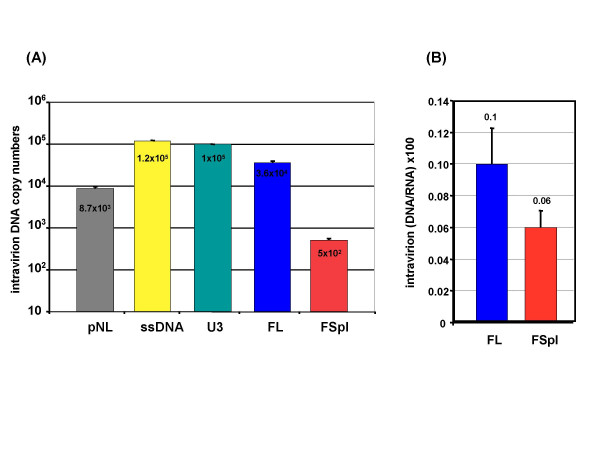
**Quantitative analysis of NERT**. (A) Intravirion DNA copy numbers quantified in virion samples corresponding to100ng of HIV p24. The DNAse treatment of virus particles lead to a plasmid DNA contamination reduced to 25%. (B) Comparison of NERT efficiencies between FL and FSpl RNAs. Results represent mean ± standard deviation.

NERT activity has been reported previously in non-permeabilized HIV-1 virions and constitutes a real potential target for the development of lentivirucides [9]. Because RTion of the FSpl RNAs might also escape the AZT inhibition through a higher NERT activity than FL RNA before cell infection, we therefore searched for HIV cDNA forms in HIV-1 particles. Thus, we compared the relative NERT efficiencies between FL and FSpl RNAs (Fig. 5A).

In these experiments, the pelleted virions were incubated with DNase at 37°c for 45 min. This step was crucial to reduce the pNL4-3 plasmid contamination under the level of the FL cDNA. First, we evaluated the abundance of the early reverse-transcribed intermediates, ssDNA and U3, that showed similar and significant intravirion amounts (Fig. 5A), demonstrating the presence of NERT activity in the HIV-1 progeny. Similar experiments were then conducted with primers specific for FL and FSpl cDNA and showed that FL RTion products were more abundant than "Fspl cDNA" forms. NERT efficiencies (Fig. 5B) were then calculated as ratios of intravirion DNA and intravirion RNA ((DNA/RNA) × 100). These results revealed that FSpl RNAs were reverse transcribed inside virions with similar efficiencies as FL RNA (0.06% and 0.1%, respectively) and definitively not with higher efficiencies.

The relative abundance of FSpl RTion products in infected cells (1 FSpl for 44 FL) favors the hypothesis of a possible role in early steps of infection. Interestingly, it was previously reported that the FL cDNA was expressed as a non-integrated form at the very beginning of infection to give FL RNA and spliced RNAs ([10,11], for review see [12] and references herein). Our results suggest that spliced RNAs could also arise from direct expression of their non-integrated cDNAs. Such early transcriptional activity from non-integrated DNAs could be highly significant in HIV-1 infection since all protein products of the FSpl RNAs are particularly crucial to engage infection. Altogether, these packaging and RTion abilities harbored by the spliced RNAs along with their low sensitivity towards RTion inhibitor, could bring first elements to explain the recent observation of high amounts of FSpl RNAs in virions isolated from plasma of AIDS patients under highly active antiretroviral therapy [13]. Indeed, the selection mediated by RTion inhibitors used to treat patients could lead to increased proportion of spliced forms of HIV RNAs and cDNAs, and consequently to unusual encapsidation levels. This could increase recombination events between spliced and FL DNAs [5]. These results should also be considered with respect to gene therapy protocols that commonly used HIV-producer cells with replication-defective vectors derived from HIV [14]. Indeed, in these systems using Psi-minus genomic RNA, encapsidation of spliced viral RNAs is drastically enhanced [2] and subsequent RTion of those RNAs would enable the recombination between FL and spliced forms and thus the infectious HIV recovery.

## Abbreviations

HIV-1, human immunodeficiency virus type 1, FL, full-length, FSpl, fully spliced, cps, copy numbers,, RTion, reverse transcription, RT, reverse transcriptase, AZT, Zidovudine, NERT, natural endogenous reverse transcription.

## Competing interests

The author(s) declare that they have no competing interests.

## Authors' contributions

LH and MM have conceived the study and analyzed data. LH performed the laboratory work with ZM help, and helped in drafting and finalizing the manuscript. MM wrote the manuscript. All authors read and approved the final manuscript.

## References

[B1] Muriaux D, Rein A (2003). Encapsidation and transduction of cellular genes by retroviruses. Front Biosci.

[B2] Houzet H, Smagulova F, Paillart J, Maurel S, Morichaud Z, Marquet R, Mougel M (2007). HIV controls the selective packaging of genomic, spliced viral, and cellular RNAs into virions through different mechanisms. Nucleic Acids Res.

[B3] Gotte M, Li X, Wainberg MA (1999). HIV-1 reverse transcription: a brief overview focused on structure-function relationships among molecules involved in initiation of the reaction. Arch Biochem Biophys.

[B4] Biard-Piechaczyk M, Robert-Hebmann V, Richard V, Roland J, Hipskind RA, Devaux C (2000). Caspase-dependent apoptosis of cells expressing the chemokine receptor CXCR4 is induced by cell membrane-associated human immunodeficiency virus type 1 envelope glycoprotein (gp120). Virology.

[B5] Liang C, Hu J, Russell RS, Kameoka M, Wainberg MA (2004). Spliced human immunodeficiency virus type 1 RNA is reverse transcribed into cDNA within infected cells. AIDS Res Hum Retroviruses.

[B6] Papadopulos-Eleopulos E, Turner VF, Papadimitriou JM, Causer D, Alphonso H, Miller T (1999). A critical analysis of the pharmacology of AZT and its use in AIDS. Curr Med Res Opin.

[B7] Quan Y, Liang C, Inouye P, Wainberg MA (1998). Enhanced impairment of chain elongation by inhibitors of HIV reverse transcriptase in cell-free reactions yielding longer DNA products. Nucleic Acids Res.

[B8] Quan Y, Rong L, Liang C, Wainberg MA (1999). Reverse transcriptase inhibitors can selectively block the synthesis of differently sized viral DNA transcripts in cells acutely infected with human immunodeficiency virus type 1. J Virol.

[B9] Zhang H, Dornadula G, Pomerantz RJ (1998). Natural endogenous reverse transcription of HIV type 1. AIDS Res Hum Retroviruses.

[B10] Brussel A, Sonigo P (2004). Evidence for gene expression by unintegrated human immunodeficiency virus type 1 DNA species. J Virol.

[B11] Wu Y, Marsh JW (2001). Selective transcription and modulation of resting T cell activity by preintegrated HIV DNA. Science.

[B12] Wu Y (2004). HIV-1 gene expression: lessons from provirus and non-integrated DNA. Retrovirology.

[B13] Saurya S, Lichtenstein Z, Karpas A (2005). Defective rev response element (RRE) and rev gene in HAART treated AIDS patients with discordance between viral load and CD4+ T-cell counts. J Clin Virol.

[B14] Sinn PL, Sauter SL, McCray PB (2005). Gene therapy progress and prospects: development of improved lentiviral and retroviral vectors – design, biosafety, and production. Gene Ther.

